# Hypocholesterolaemic Activity of Lupin Peptides: Investigation on the Crosstalk between Human Enterocytes and Hepatocytes Using a Co-Culture System Including Caco-2 and HepG2 Cells

**DOI:** 10.3390/nu8070437

**Published:** 2016-07-22

**Authors:** Carmen Lammi, Chiara Zanoni, Simonetta Ferruzza, Giulia Ranaldi, Yula Sambuy, Anna Arnoldi

**Affiliations:** 1 Department of Pharmaceutical Sciences, University of Milan, Milan I-20133, Italy; carmen.lammi@unimi.it (C.L.); chiara.zanoni@guest.unimi.it (C.Z.); 2 CREA, Food and Nutrition Research Centre, Rome I-00100, Italy; simonetta.ferruzza@crea.gov.it (S.F.); giulia.ranaldi@crea.gov.it (G.R.); yula.sambuy@crea.gov.it (Y.S.)

**Keywords:** bioactive food peptides, cholesterol metabolism, intestinal absorption, LDL receptor, Lupinus, PCSK9

## Abstract

Literature indicates that peptic and tryptic peptides derived from the enzymatic hydrolysis of lupin protein are able to modulate cholesterol metabolism in human hepatic HepG2 cells and that part of these peptides are absorbed in a small intestine model based on differentiated human Caco-2 cells. In this paper, a co-culture system, including Caco-2 and HepG2 cells, was investigated with two objectives: (a) to verify whether cholesterol metabolism in HepG2 cells was modified by the peptides absorption through Caco-2 cells; (b) to investigate how lupin peptides influence cholesterol metabolism in Caco-2 cells. The experiments showed that the absorbed peptides, not only maintained their bioactivity on HepG2 cells, but that this activity was improved by the crosstalk of the two cells systems in co-culture. In addition, lupin peptides showed a positive influence on cholesterol metabolism in Caco-2 cells, decreasing the proprotein convertase subtilisin/kexin type 9 (PCSK9) secretion.

## 1. Introduction

Lupin seed is gaining increasing attention due to its nutritional features, in particular the abundance of protein with a favourable essential amino acid composition [1], unsaturated fatty acids, fibre, minerals, carotenoids, tocoferols [2], and polyphenols [3], whereas isoflavones content is negligible [4]. Moreover, different studies have shown that lupin consumption provides useful health benefits [5], particularly in the area of hyperglycaemia prevention [6,7], hypertension control [8,9], and cholesterol reduction. This activity has been investigated in animal models, such as rat [10,11], rabbit [12], and hamster [13], and in clinical trials [14,15].

In particular, a study performed in human hepatic HepG2 cells has provided information on the lupin seed components responsible for the hypocholesterolaemic activity and the molecular mechanisms involved [16]. Tryptic and peptic peptides obtained by enzymatic hydrolysis of lupin protein inhibit the activity of 3-hydroxy-3-methylglutaryl coenzyme A reductase (HMGCoAR), up-regulate the low density lipoprotein receptor (LDLR) and sterol regulatory element binding protein 2 (SREBP-2), and increase the LDL-uptake. In addition, other evidences suggest that lupin protein can modulate the production of proprotein convertase subtilisin/kexin type 9 (PCSK9), a protein which is correlated to atherosclerosis progression and is, therefore, a novel target for hypocholesterolaemic drugs [17,18,19,20]. In fact, after consuming dietary bars containing lupin protein (30 g) for four weeks, mild hypercholesterolaemic subjects showed an 8.5% decrease of plasma PCSK9 levels, positively associated with lower levels of total plasma cholesterol, compared to the control subjects who consumed bars containing casein (30 g) [21]. Moreover, in vitro experiments performed on HepG2 cells have demonstrated that peptic and tryptic peptides obtained by hydrolysis of lupin protein positively affect the LDLR pathway, reducing PCSK9 protein levels and secretion as well as inhibiting hepatic nuclear factor 1 alpha (HNF-1 alpha) [21].

Intestinal bioavailability is critical for dietary bioactive compounds and can be investigated in vitro by conducting trans-epithelial transport experiments across a monolayer of differentiated human intestinal Caco-2 cells. In fact, these cells spontaneously differentiate in culture, expressing several morphological and functional characteristics of mature small intestinal enterocytes [22,23]. This intestinal model has extensively been used for investigating the absorption of different food components, peptides included [24]. To investigate the absorption of lupin peptides, in a previous work Caco-2 cells differentiated in a two-compartment system were treated with peptic or tryptic lupin peptides in the apical (AP) compartment, and the peptides passing in the basolateral (BL) compartment were analyzed and shown to express a moderately improved inhibitory activity on the catalytic site of HMGCoAR compared to the starting peptide mixtures [25].

**Figure 1:**
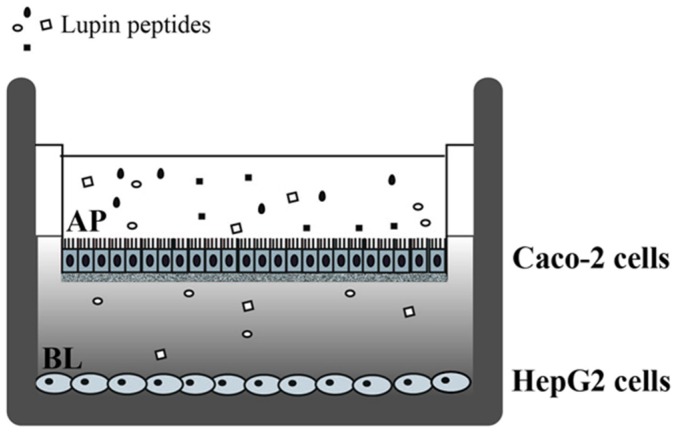
Schematic representation of the co-culture model. Caco-2 cells were grown and differentiated on polycarbonate filter membranes for 21 days. Confluent human hepatic HepG2 cells were seeded in 6-wells culture plates in complete medium. For co-culture experiments, filter inserts with differentiated Caco-2 cells were transferred to wells containing hepatic cell cultures and treated with lupin peptides for 24 h in the apical chamber. AP: Apical side, BL: Basolateral side.

In this context, the main goal of the present work was to evaluate whether the lupin peptides absorbed across Caco-2 cells still maintain their hypocholesterolaemic effect on hepatocytes, i.e., the major cells involved in the clearance of plasma LDL cholesterol [26,27]. To achieve this objective, human intestinal cells and human hepatic cells were combined in a co-culture model (Figure 1), in order to reproduce the tissue crosstalk occurring in vivo.

HepG2 cells were selected as the hepatic cell line, since the same model had been previously used for the characterization of the molecular mechanism through which tryptic and peptic lupin peptide mixtures mediate their hypocholesterolaemic effects [16]. Another goal of this work was to verify whether lupin peptides could affect cholesterol metabolism in differentiated Caco-2 cells, by specifically investigating the modulation of the LDLR-SREBP2 pathway and PCSK9 production. In fact, there is increasing evidence that, in addition to being involved in the uptake of cholesterol, the small intestine can also actively secrete this molecule, with a mechanism named trans-intestinal cholesterol efflux (TICE) pathway [28], which contributes in a significant way to the total fecal neutral sterol excretion together with the better-known hepatobiliary route [29,30]. Although further research is necessary for a better understanding of the mechanistic details of the TICE pathway, its therapeutic potential in the treatments of atherosclerosis appears to be encouraging [28]. The Caco-2 cell model is appropriate for investigating the in vitro effects of lupin peptides on the LDLR/SREBP2 pathway in the intestine.

## 2. Materials and Methods

### 2.1. Chemicals and Reagents

Dulbecco’s modified Eagle’s medium (DMEM) was bought from GIBCO (Thermo Fisher Scientific, Waltham, MA, USA). Fetal bovine serum (FBS) was from Hyclone Laboratories (Logan, UT, USA). Stable l-glutamine, 1% non-essential amino acids, penicillin/streptomycin, and chemiluminescent reagent were from Euroclone (Milan, Italy). Polycarbonate filters, 12 mm diameter, 0.4 µm pore diameter were from Transwell Corning Inc. (Lowell, MA, USA). Phenol red, PBS, bovine serum albumin (BSA), RIPA buffer, and the antibody against β-actin were from Sigma-Aldrich (St. Louis, MO, USA). The antibody against HMGCoAR was bought from Abcam (Cambridge, UK), that against PCSK9 from GeneTex (Irvine, CA, USA), that against phospho-HMGCoAR (Ser872) from Bioss Antibodies (Woburn, MA, USA), and that against LDLR from Pierce (Rockford, IL, USA). The antibodies against SREBP-2, rabbit Ig-HRP, mouse Ig-HRP, phenylmethanesulfonyl fluoride (PMSF), Na-orthovanadate inhibitors were purchased from Santa Cruz Biotechnology Inc. (Santa Cruz, CA, USA); the inhibitor cocktail Complete Midi from Roche (Basel, Switzerland). Mini protean TGX pre-cast gel 7.5% and Mini nitrocellulose Transfer Packs were purchased from BioRad (Hercules, CA, USA). The human proprotein convertase 9 immunoassay (Quantikine ELISA) was bought from R & D System (Minneapolis, MN, USA).

### 2.2. Preparation of Peptic and Tryptic Peptide Mixtures

Lupin seeds (*Lupinus albus* cultivar Ares) were provided by Terrena (Matrignè-Ferchaud, France). Procedures for the preparation of total protein extract, hydrolysis of the protein with pepsin or trypsin to produce peptic and tryptic peptides, and analytical method by nano-HPLC-ESI-MS/MS have been previously reported [16,21].

### 2.3. Cell Culture and Differentiation

Caco-2 cells, obtained from INSERM (Paris) were routinely sub-cultured at low density (50%) [31] and maintained at 37 °C in a 90%/10% air/CO_2_ atmosphere in DMEM containing 25 mM glucose, 3.7 g/L NaHCO_3_, 4 mM stable l-glutamine, 1% non-essential amino acids, 100 U/L penicillin, 100 µg/L streptomycin (complete medium), supplemented with 10% heat inactivated fetal bovine serum (FBS) (Hyclone Laboratories, Logan, UT, USA). For differentiation, cells were seeded on polycarbonate filters, 12 mm diameter, 0.4 µm pore diameter (Transwell, Corning Inc., Lowell, MA, USA) at a 3.5 × 105 cells/cm^2^ density in complete medium supplemented with 10% FBS in both AP and BL compartments for 2 days to allow the formation of a confluent cell monolayer. Starting from the third day after seeding, cells were transferred to complete medium in both compartments, supplemented with 10% FBS only in the BL compartment, and allowed to differentiate for 21 days with regular medium changes three times weekly [32].

The HepG2 cell line was bought from ATCC (HB-8065, LGC Standards, Milan, Italy). The HepG2 cell line was cultured in DMEM high glucose with stable l-glutamine supplemented with 10% FBS, 100 U/mL penicillin, 100 µg/mL streptomycin (complete growth medium) and incubated at 37 °C under 5% CO_2_ atmosphere. Caco-2 and HepG2 cells were used for no more than 20 passages after thawing, as the increase in the number of passages may change the cell characteristics and impair assay results.

### 2.4. Cell Treatments with Lupin Peptides

The treatments with lupin peptides were conducted on 21-days differentiated intestinal Caco-2 cells, alone or in co-culture with HepG2 cells at the bottom of the culture plate (Figure 1). For co-culture experiments, Caco-2 cells on filter inserts were transferred in multiwell culture plates containing confluent HepG2 cells. Prior to treatment with lupin peptides, differentiated Caco-2 cells were washed twice with 500 µL PBS with 1 mM Ca^2+^ and 1 mM Mg^2+^. The peptic or tryptic digests of lupin protein (1.0 µg/µL) were added in the complete medium (500 µL) of the AP compartment, whereas the BL compartment contained complete medium supplemented with 10% FBS (700 µL). After 24 h incubation of cells alone or in co-culture, AP and BL media and all cells were collected for further analysis. Three independent experiments were conducted either on intestinal Caco-2 cells alone or in co-culture, each in duplicate. The concentration of the peptides in the AP and BL solutions were determined as indicated in a previous paper [25].

### 2.5. Cell Monolayer Integrity and Differentiation Evaluation

In order to evaluate the degree of Caco-2 cell differentiation and the integrity of the cell monolayer, trans-epithelial electrical resistance (TEER) was measured at 37 °C using the voltmeter apparatus Millicell (Merck Millipore Co., Darmstadt, Germany), immediately before and at the end of 24 h incubation with the tryptic and peptic peptides. After peptides incubation, no significant changes in TEER values were observed.

### 2.6. Western Blot Analysis

After 24 h incubation, Caco-2 cells and, in co-culture experiments, also HepG2 cells were scraped in 100 µL of ice-cold lysis buffer (RIPA buffer + inhibitor cocktail + 1:100 PMSF + 1:100 Na-orthovanadate) and transferred in ice-cold microcentrifuge tubes. After centrifugation at 16,060 *g* for 15 min at 4 °C, the supernatant was recovered and transferred in a new ice-cold tube. Total proteins were quantified by the Bradford method and 50 μg of total proteins loaded on a pre-cast 7.5% sodium dodecyl sulphate—polyacrylamide (SDS-PAGE) gel at 130 V for 45 min. Subsequently, the gel was transferred to a nitrocellulose membrane (Mini nitrocellulose Transfer Packs), using a Trans-blot Turbo at 1.3 A, 25 V for 7 min. Target proteins, on milk blocked membrane, were detected by primary antibodies as follows: anti-SREBP2, anti-LDLR, anti-HMGCoAR, anti-phospho-HMGCoAR (Ser872), anti-PCSK9, and anti-β-actin. Secondary antibodies conjugated with HRP, a chemiluminescent reagent, were used to visualize target proteins, and their signal was quantified using the Image Lab Software (Biorad, Hercules, CA, USA). The internal control β-actin was used to normalize loading variations.

### 2.7. Quantification of Excreted PCSK9 in Cell Culture Experiments by ELISA

The AP and BL media collected from treated Caco-2 cells were centrifuged at 600 *g* for 10 min at 4 °C, then recovered in a new ice-cold tube. PCSK9 was quantified by ELISA (R & D System). The lower limit of detection was 0.096 ng/mL

### 2.8. Statistically Analysis

Statistical analyses were carried out by one-way ANOVA using the software Prism 6 (GraphPad, La Jolla, CA, USA) followed by Dunnett’s test. Values were expressed as means ± SEM; *p*-values < 0.05 were considered to be significant.

## 3. Results

### 3.1. Absorbed Lupin Peptides Maintain the Capacity to Induce the Up-Regulation of LDLR-SREBP2 Pathway in HepG2 Cells

**Figure 2:**
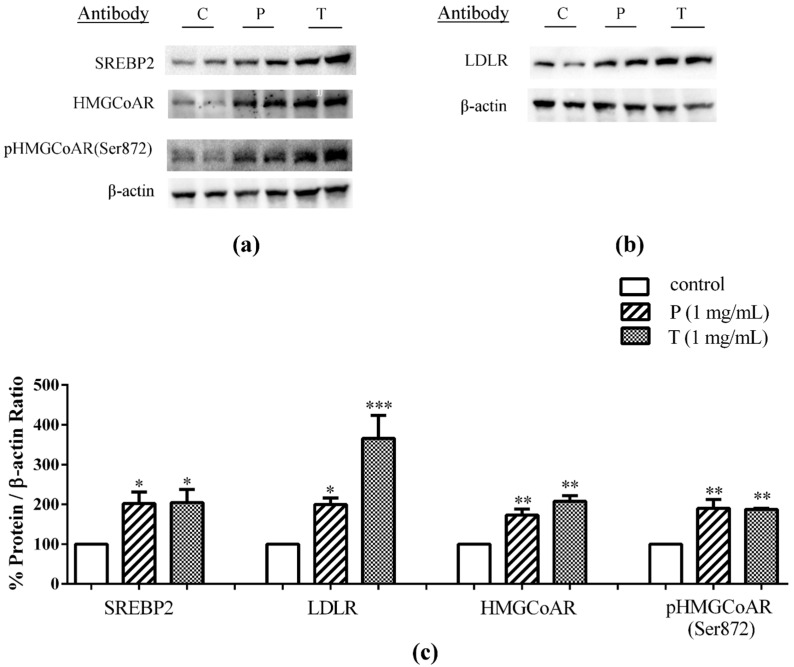
After differentiation on polycarbonate filter membranes for 21 days, Caco-2 cells were transferred on HepG2 cells and human intestinal cells were treated with 1.0 μg/μL of peptic peptides (P) or tryptic peptides (T) for 24 h, respectively. The second day, HepG2 cells in the co-culture system were harvested and processed for immunoblotting experiments. SREBP-2 (**a**), HMGCoAR (**a**), pHMGCoAR (Ser872) (**a**), LDLR (**b**) and β-actin (**a**,**b**) immunoblotting signals were detected using specific anti-SREBP-2, anti-HMGCoAR, anti-pHMGCoAR (Ser872), anti-LDLR, and anti-β-actin primary antibodies, respectively; Each protein signal was quantified by ImageLab software (Biorad) and normalized with β-actin signals (**c**). Bars represent averages of duplicate samples ± SEM of three independent experiments. (*) *p* < 0.05, (**) *p* < 0.01 and (***) *p* < 0.001 versus untreated sample (C).

In a previous paper we have demonstrated that when digested lupin proteins are applied to the AP surface of a monolayer of differentiated intestinal Caco-2 cells, a certain number of peptides are transferred intact to the BL compartment and these peptide mixtures retain the ability to inhibit HMGCoA in vitro activity [25]. In order to evaluate whether the absorbed peptides maintain their capacity to modulate cholesterol metabolism in hepatocytes, a co-culture system was set up combining differentiated Caco-2 cells cultured on filter inserts and hepatic HepG2 cells grown at the bottom of the culture plates (Figure 1). After treating the intestinal cells with 1.0 μg/μL peptic or tryptic peptides from the AP side for 24 h, the underlying HepG2 cells in co-culture were harvested to assess by immunoblotting the expression of target proteins. Figure 2a–c shows that the treatment of intestinal cells with both peptide mixtures induced an up-regulation of the protein level of SREBP2 N-terminal fragment (mature form with a molecular weight of 68 kDa) in the underlying HepG2 cells in co-culture. In particular, peptic peptides up-regulated the mature SREBP2 protein level by 102% versus the untreated cells and tryptic peptides by 104%. In the same experiments, both peptic and tryptic peptides increased LDLR and HMGCoAR protein levels, in agreement with their capacity to up-regulate the level of mature-SREBP2 protein. LDLR protein levels were increased by 100% with peptic peptides and by 266% with tryptic peptides versus the untreated cells (Figure 2b,c), whereas peptic peptides enhanced the production of the HMGCoAR protein by 73% and tryptic peptides by 108% versus the untreated cells (Figure 2a,c). Finally, both peptide digests increased the inactive phosphorylated form of HMGCoAR, i.e., p-HMGCoAR: peptic peptides by 90% and tryptic peptides by 87% versus untreated cells, respectively (Figure 2a,c).

### 3.2. Lupin Peptides Mediate the Up-Regulation of LDLR-SREBP2 in Human Intestinal Cells

**Figure 3:**
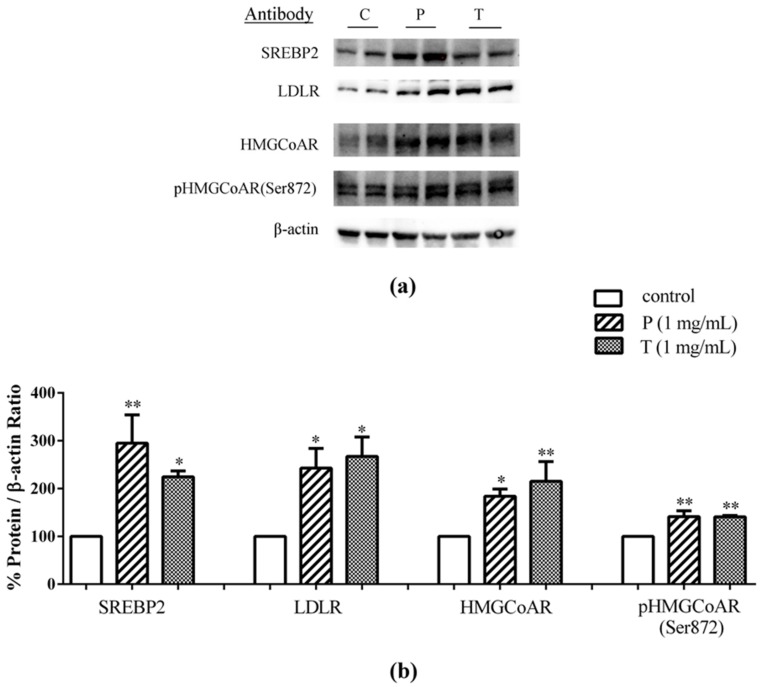
After differentiation on polycarbonate filter membranes for 21 days, Caco-2 cells were transferred on HepG2 cells and human intestinal cells were treated with 1.0 μg/μL of peptic peptides (P) or tryptic peptides (T) for 24 h, respectively. The second day, treated Caco-2 cells were harvested and processed for immunoblotting experiments. SREBP-2, LDLR, HMGCoAR, pHMGCoAR (Ser872), and β-actin immunoblotting signals were detected using specific anti-SREBP-2, anti-LDLR, anti-HMGCoAR, anti-pHMGCoAR (Ser872), and anti-β-actin primary antibodies, respectively (**a**); Each protein signal was quantified by ImageLab software (Biorad) and normalised with β-actin signals (**b**). Bars represent averages of duplicate samples ± SEM of three independent experiments. (*) *p* < 0.05 and (**) *p* < 0.01 versus untreated sample (C).

To assess whether lupin peptides in the same co-culture system could also modulate cholesterol pathways in the enterocytes, differentiated Caco-2 cells were treated with peptic and tryptic lupin digests in the AP compartment for 24 h. Immunoblotting experiments showed that this treatment induced increments of the protein level of SREBP2 N-terminal fragment (mature form with a molecular weight of 68 kDa), of 195% and 125%, versus the untreated cells, for peptic and tryptic lupin peptides, respectively (Figure 3a,b). In addition, increased LDLR and HMGCoAR protein levels were observed: Peptic and tryptic peptides up-regulated LDLR by 143% and 167%, respectively, versus the untreated cells, and they increased HMGCoAR by 84% and 115%, respectively, versus the control. Finally, peptic and tryptic peptides increased also the inactive phosphorylated form of HMGCoAR by 41.3% and 40.9%, respectively, versus the untreated cells.

**Figure 4:**
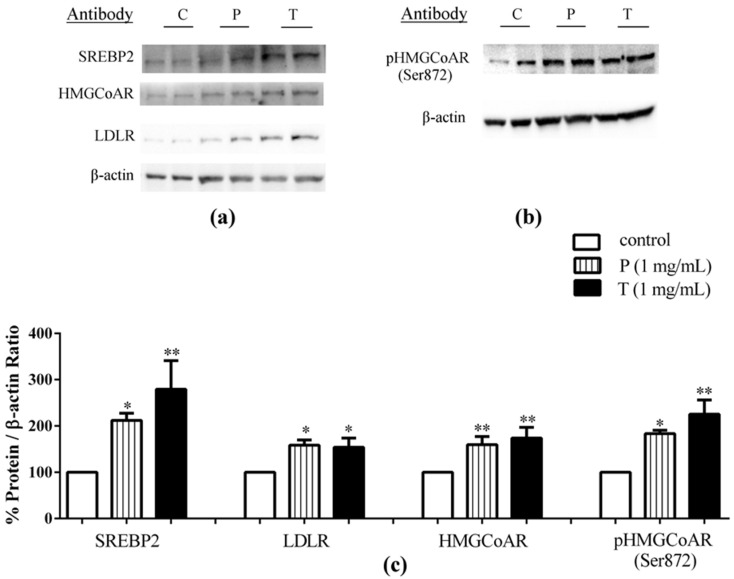
After differentiation on polycarbonate filter membranes for 21 days, Caco-2 cells were treated with 1.0 μg/μL of peptic peptides (P) or tryptic peptides (T) for 24 h, respectively. The second day, Caco-2 cells were harvested and processed for immunoblotting experiments. SREBP-2 (**a**), LDLR (**a**), HMGCoAR (**a**), pHMGCoAR (Ser872) (**b**), and β-actin (**a**,**b**) immunoblotting signals were detected using specific anti-SREBP-2, anti-LDLR, anti-HMGCoAR, anti-pHMGCoAR (Ser872), and anti-β-actin primary antibodies, respectively; Each protein signal was quantified by ImageLab software (Biorad) and normalised with β-actin signals (**c**). Bars represent averages of duplicate samples ± SEM of three independent experiments. (*) *p* < 0.05 and (**) *p* < 0.01 versus untreated sample (C).

For comparison, other experiments were performed to evaluate the effects of lupin peptide treatments on cholesterol metabolism pathway in Caco-2 cells cultured alone (Figure 4). The findings were similar to those obtained in the co-culture system. Peptic and tryptic peptides up-regulated mature SREBP2 protein levels by 112%, and 179%, respectively (Figure 4a–c), and LDLR protein by 58% and 54%, respectively, versus the untreated cells (Figure 4a–c). Increases of HMGCoAR protein levels by 60% after peptic and by 74% after tryptic peptides treatment, versus the control, were also observed (Figure 4a–c), accompanied by concomitant increases of the inactive p-HMGCoAR form by 83% for peptic and by 125% for tryptic peptides, versus the untreated cells (Figure 4b,c).

### 3.3. Lupin Peptides Decrease the Ability of Caco-2 Cells to Secrete PCSK9 in the BL Medium

**Figure 5:**
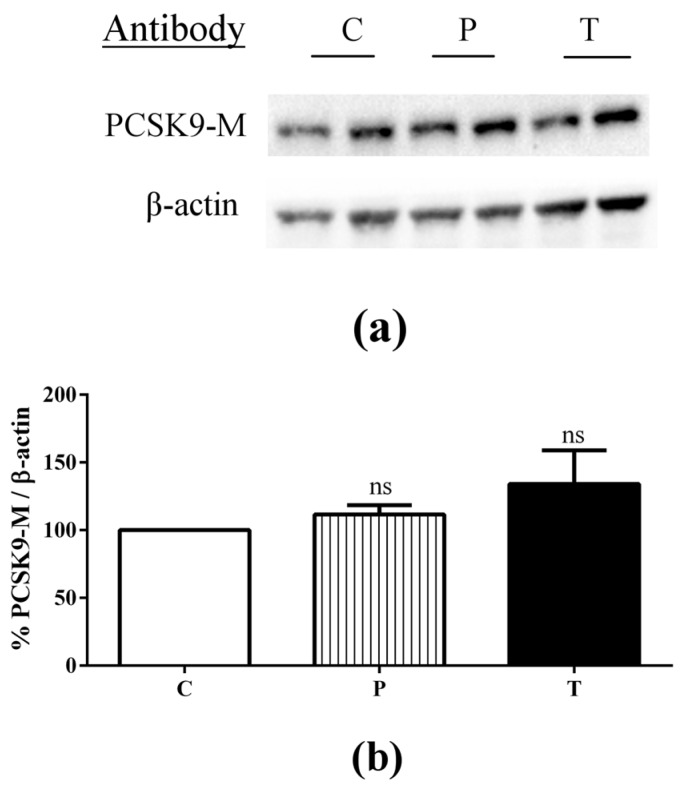
Immunoblotting experiments were carried out in order to assess whether peptic and tryptic lupin peptides are able to affect the PCSK9 protein levels. After differentiation on polycarbonate filter membranes for 21 days, Caco-2 cells alone were treated with 1.0 μg/μL of peptic peptides (P) or tryptic peptides (T) for 24 h, respectively. The second day, Caco-2 cells were harvested and processed for immunoblotting experiments (**a**,**b**). Mature PCSK9 (PCSK9-M) and β-actin immunoblotting signals were detected using specific anti-PCSK9-M and anti-β-actin primary antibodies, respectively (**a**); Each protein signal was quantified by ImageLab software (Biorad) and normalised with β-actin signals (**b**). Bars represent averages of duplicate samples ± SEM of three independent experiments. Ns: not significant.

In Caco-2 cells cultured alone, immunoblotting and ELISA experiments were carried out in order to assess whether peptic and tryptic lupin peptides were able to affect the levels of intracellular and secreted PCSK9 in enterocytes. Intracellular PCSK9 protein levels in Caco-2 cells were not significantly affected by 24-h incubation with peptic and tryptic lupin peptides in the AP compartment (Figure 5a,b).

In order to evaluate whether the secretion of mature PCSK9 was affected by lupin peptides treatment, and in which direction, its concentration was measured by a specific ELISA in the AP and BL media at the end of the experiment. Lupin peptides treatment resulted in a significant reduction in the secretion of mature PCSK9 in the BL compartment, versus untreated cells (Table 1). More in detail, untreated Caco-2 cells secreted 5.0 ng/mL of mature PCSK9, whereas those treated with peptic peptides secreted 4.1 ng/mL PCSK9 and those treated with tryptic lupin peptides secreted 3.2 ng/mL PCSK9, respectively. Conversely, no mature PCSK9 secretion was detected in the AP compartment, since PCSK9 levels were, in all cases, below the lower limits of detection of the assay (i.e., 0.096 ng/mL).

## 4. Discussion

### 4.1. Absorbed Lupin Peptides Maintain Their Hypocholesterolaemic Activity on Human Hepatic HepG2 Cells Grown in a Co-Culture System

A preceding work [25] has provided evidence that lupin peptides transported from the AP to the BL compartment in Caco-2 cells maintain intact their initial capacity of inhibiting the enzymatic activity of HMGCoAR. This has stimulated us to evaluate whether the absorbed lupin peptides were still able to modulate cholesterol metabolism in hepatic cells, by utilizing a co-culture system, in which human enterocytes and hepatocytes were combined in order to reproduce as far as possible the tissue crosstalk occurring in vivo. Indeed, results indicated that the treatment of Caco-2 cells with lupin peptides in the AP compartment produced a favorable modulation of the LDLR pathway in HepG2 cells cultured at the bottom of the BL compartment. In fact, absorbed peptides increased mature SREBP2 protein level, with the consequent up-regulation of LDLR and HMGCoAR protein levels. Whereas in the case of peptic peptides, the values observed in the co-culture system were slightly lower than those in isolated HepG2 cells, i.e., SREBP-2 148%, LDLR 136%, HMGCoAR 73% [16], surprisingly, in the case of tryptic peptides, the values observed in the co-culture system indicated an improvement of LDLR pathway activation compared to the values observed in isolated Hep2 cells, i.e., SREBP-2 73%, LDLR 84%, HMGCoAR 97% [16]. Although, the peptide mixture was, in the case of the co-culture system, selectively “filtered” by the passage across the Caco-2 cell monolayer, while it was unmodified when directly applied to HepG2 cultured alone, these comparisons highlight important differences in the activity of absorbed peptides form lupin digestion. These results suggest in fact a selective absorption of more active peptides from the tryptic digest, favoring a stronger positive modulation of cholesterol metabolism.

**Figure A1:**
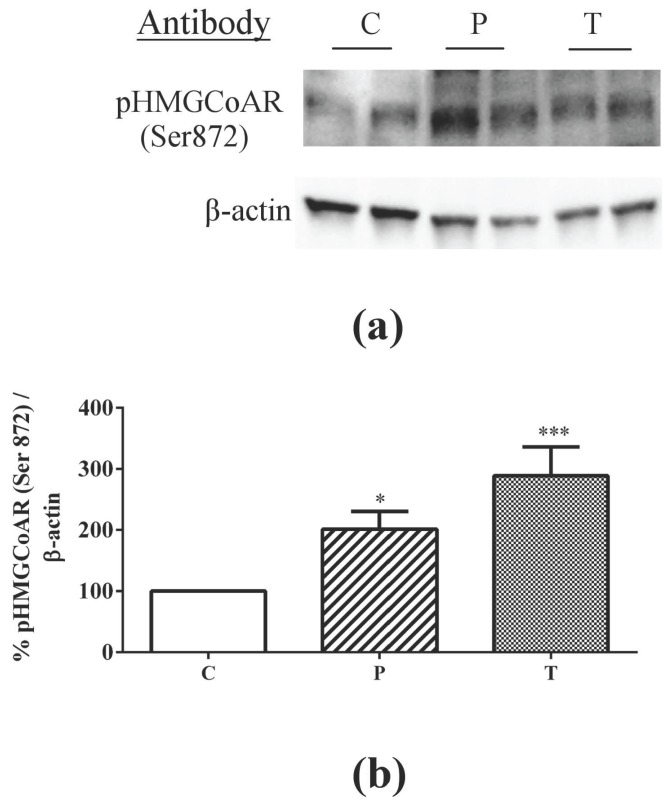
Effects of peptic and tryptic lupin peptides on the phosphorylation level of HMGCoAR in HepG2 cells. (**a**) Western blots; (**b**) percentage change of pHMGCoAR protein expression after treatment with peptic peptides (P) or tryptic peptides (T), C = control treatment.

Finally, it is important to observe that the up-regulation of the HMGCoAR protein level is not expected to exert a negative effect on cholesterol metabolism, since it can be accounted for by the increase of the inactive Ser872-phosphorylated species (Figure 2). This is in agreement with the effects observed in HepG2 cells cultured alone, where the direct treatment with lupin peptides (1.0 μg/μL) induced an increase in the enzymatically inactive phosphorylated form of HMGCoAR (Ser872) by 101% with peptic and by 190% with tryptic digests, versus untreated cells (Figure A1 in Appendix materials).

### 4.2. Role of Lupin Peptides in the Regulation of Cholesterol Metabolism in Human Intestinal Cells

Serum LDL levels are determined by a balance between rates of production and clearance of LDLs from the circulation, where not only the liver, but also the intestine plays a key role [28]. For this reason, more and more attention is currently paid to the potential health benefits of lowering intestinal lipid absorption, in view of the impact on postprandial lipoprotein metabolism and atherosclerosis [33], although the complex molecular mechanisms underlying cholesterol homeostasis in the small intestine have not been completely elucidated yet.

The present study provides evidence that lupin peptides are able to up-regulate LDLR through activation of SREBP2 in intestinal cells either cultured alone or in co-culture system in temporal and spatial proximity with human hepatic HepG2 cells. Figure 3 and Figure 4 indicate that the treatment of Caco-2 cells with lupin peptides produced slightly better effects on the LDLR pathway in the co-culture system than when Caco-2 cells were treated alone. These findings suggest that the cross talk between the intestine and hepatic system can improve the effects of lupin peptides on cholesterol metabolism through LDLR protein up-regulation.

The LDLR expression and localization at the cellular membranes are strictly correlated with the pathway of cholesterol biosynthesis. In fact, the transcription of LDLR and the genes required for cholesterol and fatty acid synthesis are controlled by membrane-bound transcription factors called SREBPs [34] and the levels of intracellular cholesterol act through a negative feedback inhibition mechanism [35]. The SREBP2 isoform is responsible for LDLR and HMGCoAR transcription and SREBP2 maturation is regulated by intracellular cholesterol homeostasis. Thus, LDLR up-regulation in intestinal cells may represent a useful strategy to contribute to the control of plasma LDL cholesterol levels. Different studies have established that the LDLR is predominantly localized at the BL membrane of polarized well differentiated Caco-2 cells [36,37], in agreement to its localization in mature enterocytes.

In accordance with SREBP2 activation, both peptic and tryptic lupin peptides determined an increase of HMGCoAR protein levels. This enzyme is among the most highly regulated ones [38], since it is long-term regulated by the control of its synthesis and degradation and short-term regulated through phosphorylation or dephosphorylation [39]. In particular, the phosphorylation of Ser872 by 5′-adenosine monophosphate-activated protein kinase (AMPK) decreases the enzyme activity [40]. The immunoblotting results indicate that lupin peptides induce an increase of the inactive phosphorylated form of HMGCoAR that can account for most of the observed increase in total HMGCoAR induced by lupin peptides (Figure 4b,c).

Numerous studies have pointed out the importance of the regulation of LDLR by PCSK9 in the liver [41], which has emerged as a promising target for new cholesterol-lowering therapy. In this context, we have recently demonstrated that peptic and tryptic lupin peptides are able to decrease the PCSK9 protein levels as well as to reduce the HNF1-alpha protein levels in human hepatic HepG2 cells [21]. Stimulated by some recent studies underlining the importance of PCSK9 in the intestine [36], in this work, we have investigated the effects of lupin peptides treatment on the production of PCSK9 in Caco-2 cells. In particular, recent studies performed on Caco-2 cells underline that PCSK9 play an important role in intestinal triglyceride-rich apoB lipoprotein production [42] and postprandial lipaemia [43]. Moreover, recent evidences clearly suggest that PCSK9 is implicated in the modulation of TICE [30]. The ability of intestinal cells to acquire cholesterol is modulated in part by Niemann-Pick C1-Like 1 (NPC1L1) mediated luminal cholesterol uptake and, on the BL side, by the uptake of LDL particles, a process dependent on LDLR activity. Notably, PCSK9−/− mice were shown to have increased fecal cholesterol excretion, whereas intravenous injection of recombinant PCSK9 acutely in these mice reduced TICE by 35% [44]. Conversely, PCSK9 administration had no effect on TICE in LDLR knockout mice. This observation, together with the finding that wild-type mice treated with lovastatin for 10 days experienced a 71% increase in TICE, provides evidence that TICE modulation by PCSK9 is dependent on LDLR expression [44]. Although the functional role of intestinal LDLR in lipoprotein metabolism remains to be established, these findings suggest that targeting PCSK9 holds promise for the amelioration of postprandial hypertriglyceridaemia. In line with this evidence, lupin peptides decreased the secretion of PCSK9 in the BL medium, i.e., on the side where the LDLR proteins are located. This preferential BL delivery of PCSK9 suggests that this protein may directly affect the LDLR, even if the reduction of mature PCSK9 secretion was achieved without altering intracellular PCSK9 protein levels.

## 5. Conclusions

In conclusions, this work has implemented a detailed investigation on the favorable effects of lupin peptides on cholesterol metabolism in enterocytes and in a co-culture system including enterocytes and hepatocytes. The Caco-2 cell model has proved appropriate for investigating the in vitro effects of lupin peptides on the LDLR/SREBP2 pathway in the intestine. In addition, the co-culture system has allowed a more physiological approach to the study of the response of hepatocytes to molecules coming from the diet, taking into account the selective effects of transport and metabolism across the intestinal mucosa. To the best of our knowledge, this is the first application of this co-culture system to investigate transport and metabolic effects of peptides deriving from plant protein hydrolysis.

## Figures and Tables

**Table 1:** PCSK9 secreted by differentiated Caco-2 cells treated with lupin peptides (1.0 µg/µL) in the AP chamber, quantified by ELISA (LOD, limit of detection = 0.096 ng/mL).

Parameter	C	Peptic Peptides	Tryptic Peptides
Secreted PCSK9 in BL solution (ng/mL)	4.82 ± 0.35	4.10 ± 0.46 *	3.20 ± 0.25 **
Secreted PCSK9 in AP solution (ng/mL)	<LOD	<LOD	<LOD

* *p* < 0.05 vs. untreated sample (C); ** *p* < 0.01 vs. untreated sample (C).

## Abbreviations

The following abbreviations are used in this manuscript:

AMPK	5′-adenosine monophosphate-activated protein kinase
AP	apical
BL	basolateral
BSA	bovine serum albumin
DMEM	Dulbecco’s modified Eagle’s medium
FBS	Foetal bovine serum
HMGCoAR	3-hydroxy-3-methylglutaryl coenzyme A reductase
HNF-1	alpha hepatic nuclear factor 1 alpha
LDL	low density lipoprotein
LDLR	low density lipoprotein receptor
NPC1L1	Niemann-Pick C1-Like 1
PCSK9	proprotein convertase subtilisin/kexin type 9
SREBP-2	sterol regulatory element binding proteins 2
SDS-PAGE	sodium dodecyl sulphate—polyacrylamide
TEER	trans-epithelial electrical resistance
TICE	trans-intestinal cholesterol efflux
